# Challenges and barriers to improving care of the musculoskeletal patient of the future - a debate article and global perspective

**DOI:** 10.1186/1754-9493-5-23

**Published:** 2011-09-25

**Authors:** Hangama C Fayaz, Jesse B Jupiter, Hans Christoph Pape, R Malcolm Smith, Peter V Giannoudis, Christopher G Moran, Christian Krettek, Karl J Prommersberger, Michael J Raschke, Javad Parvizi

**Affiliations:** 1 Department of Orthopaedic Surgery, Harvard Medical School, Massachusetts General Hospital, 55 Fruit Street, Boston, MA 02114, USA; 2 Department of Orthopaedic Trauma, University of Aachen Medical Center, Pauwelsstraße 30, 52074 Aachen, Germany; 3 Department of Trauma and Orthopaedic Surgery, School of Medicine, University of Leeds, Leeds LS2 9JT, UK; 4 Department of Orthopaedic Trauma, University Hospital, Queen's Medical Centre, GB-Nottingham, NG7 2UH, UK; 5 Department of Trauma Surgery, Hannover Medical School, Carl-Neuberg-Str. 1, 30625 Hannover, Germany; 6 Clinic for Special Hand Surgery, Salzburger Leite 1, 97616 Bad Neustadt an der Saale, Germany; 7 Department of Trauma Surgery, University Hospital Muenster, Albert-Schweitzer-Campus 1, 48149 Münster, Germany; 8 Rothman Institute of Orthopedics Philadelphia, Thomas Jefferson University Hospital, 925 Chestnut Street, 5th Floor, Philadelphia, PA 19107, USA

**Keywords:** Global perspective, Future trends and needs, Algorithms of patient care, Quality assurance in Patient care, Registries

## Abstract

**Background:**

With greater technological developments in the care of musculoskeletal patients, we are entering an era of rapid change in our understanding of the pathophysiology of traumatic injury; assessment and treatment of polytrauma and related disorders; and treatment outcomes. In developed countries, it is very likely that we will have algorithms for the approach to many musculoskeletal disorders as we strive for the best approach with which to evaluate treatment success. This debate article is founded on predictions of future health care needs that are solely based on the subjective inputs and opinions of the world's leading orthopedic surgeons.

Hence, it functions more as a forum-based rather than a scientific-based presentation. This exposé was designed to stimulate debate about the emerging patients' needs in the future predicted by leading orthopedic surgeons that provide some hint as to the right direction for orthopedic care and outlines the important topics in this area.

**Discussion:**

The authors aim to provide a general overview of orthopedic care in a typical developed country setting. However, the regional diversity of the United States and every other industrialized nation should be considered as a cofactor that may vary to some extent from our vision of improved orthopedic and trauma care of the musculoskeletal patient on an interregional level.

In this forum, we will define the current and future barriers in developed countries related to musculoskeletal trauma, total joint arthroplasty, patient safety and injuries related to military conflicts, all problems that will only increase as populations age, become more mobile, and deal with political crisis.

**Summary:**

It is very likely that the future will bring a more biological approach to fracture care with less invasive surgical procedures, flexible implants, and more rapid rehabilitation methods. This international consortium challenges the trauma and implants community to develop outcome registries that are managed through health care offices and to prepare effectively for the many future challenges that lie in store for those who treat musculoskeletal conditions.

## Background

By 2020, in developed countries considerable modification in orthopaedic care is expected to have occurred due to changes in population demographics and innovation in operative techniques, treatments, and research. One of the most drastic changes will be due to the aging North American population. It is predicted that, by 2020, 16.3% of the US population and 25% of the Canadian population will be 65 years of age or older [1,2]. Fractures have become a leading cause of patient morbidity and mortality in both industrialized and developing countries. Fragility fractures represent a substantial public health problem, with more than 1.5 million osteoporotic fragility fractures taking place each year in the United States. In addition, by 2020, injuries caused by traffic accidents will represent the major cause of fractures. Urbanization and industrialization in China and India, which contain 40% of the world's population, has resulted in a clear increase in trauma-related injuries [3].

All of these projections indicate an increased incidence and prevalence of age- and traffic-related musculoskeletal disabilities, such as fragility fractures, osteoarthritis, and trauma-related injuries.

To minimize cost and increase orthopaedic care in the future, there should be a distinct therapeutic concept refining risk stratification, characterizing the future musculoskeletal patient, and identifying at-risk patients (Table 1).

**Table 1:** Diagnostic Concepts

Concept of developing treatment's Algorithms	
**Osteoporosis **	-Idenitifcation of Patient's phenotype
	- Patient Screening
	-Poor nutrition and exercise is an indicator for Osteoporosis
	-Defects in collagen 1a1 and 1a2 genes
	-Variations of the Wnt coreceptor; LPR5;
	
**Osteolysis and implant loosening **	-Variations in the TNF gene promoter
	-IL-1 gene family and related protein 3
	
**Monitoring patients postsurgically **	-Urine metal ion level, serum cytokine, lymphocyte response
	
**Mechanotransduction signaling and skeletal repair **	-COX2
	
**Acute systemic inflammation and bone resorption **	-TNF-α, IL-6, translocation of NF-kappa-B and TNF-alpha-mRNA expression in peripheral blood monocytes
	
**Posttraumatic stress **	-Trauma-focused cognitive-behavioral therapy
	
**Cell viability, Osteoblast differentiation, gene expression of osteoblasts **	-Level of canonical Wnt-,IGF-, TGF-β, FGF

Concept of developing treatment's Algorithms: Modified according to Cuomo AV, Lieberman JR (modified version, adapted from Ref #[6])

Screening and treatment algorithm of the future musculoskeletal patient may include the following sequence of some diagnostic and treatment module (modified according to Cuomo AV, Lieberman JR, adapted from Ref #[6]) *: at age 40 history, physical examination, MRI of all joints; genetic and proteonomic screening with microchip array; central bioinformatics database calculates up-to-date disease risks; bioengineered tissue matrix including adequate therapy concept, cartilage regeneration, tissue engineering, biologic knee replacement (meniscus replacement, articular cartilage paste grafting); minimally invasive implantation of tissue matrices; physical therapy, adjusted diet, lifestyle modification, work out modifications are prescribed; primary care prophylaxis is initiated for management of high risk conditions*.

A lowered bone mineral density is an accurate indicator of the need to begin osteoporosis treatment.

According to clinical experience, patients with osteopenia or osteoporosis that are informed about the results of their bone mineral density tend take medications and be more compliant than patients who do not know their bone mineral density results [4].

Given the potential demographic and aging changes that await us, the creation of innovative concepts for treating fractures is strongly needed.

Despite post-traumatic stress, depression, and the application of antidepressive and antipsychotic medications having a major impact on patients with fractures, one of the most neglected features of fracture healing has been the paramount importance of post-traumatic stress [5]. The ongoing US military experiences may shed more light into this important aspect of treating traumatized patients.

Screening patients for systemic risk factors (including infection, smoking, diabetes, metabolic syndrome, Factor XIII deficiency, soft tissue injury, and use of heparin, non steroidal anti inflammatory drugs and cortisol) will help to prevent and minimize risks of nonunion.

Indeed, access to information is a key element. Defining multiple risk factors enables physicians to think ahead and prevent the occurrence of multiple musculoskeletal injuries. By having access to detailed information on the osteoporosis risk index, arthroplasty loosening index and osteoarthritis risk, and considering other factors such as age, weight, and activity level, a decision specific to each patient can be made. Gene microchip arrays can also be considered as an option for rapid interhospital transfer of patient data. However, the ethical aspect of this device requires more investigation [6].

As concluded by Rozental et al. (2008), a simple intervention in the orthopaedic clinic significantly improves osteoporosis screening and treatment rates. They determined that, of the two tested interventions, the one that requires the orthopaedic surgeon to recommend a bone mineral density examination at the time of the initial fracture care visit resulted in an assessment rate (93%) that was three times higher than the rate found when this step was left to the primary care physician (30%), [4].

Microarray analysis characterizes considerable numbers of genes that demonstrate variable expression in osteoblast cultures from patients with hypertrophic nonunions compared to controls. Hoffmann et al. (2008) indicated that cell viability, osteoblast differentiation, and gene expression in osteoblasts are varied in patients that tend to develop persistent and refractory fracture nonunions. Hence, proteins included in the Wnt, insulin-like growth factor 2 (IGF2), transforming growth factor beta 2 (TGF-β2), and fibroblast growth factor (FGF) signaling pathways are of paramount importance and can guide new ideas for clinical treatments in the future [7]. However, screening and risk identification for patients may initiate several conflicting dilemmas, such as the potential for characterizing a "patient's unique genetic blueprint" and indicating his or her predisposition to various disorders. Such screening will cause health, privacy, and ethical conflicts.

Because most hypertrophic nonunions might be the result of poorly conceived and executed internal fixation, improved education and better standardization of surgical, or non-operative, techniques might possibly be far more effective at improving outcomes while controlling costs.

### Algorithm of managing trauma patients - a uniform concept

Synchronization of trauma patient assessment and management has long been an aim of trauma surgeons. Transport between facilities, or even within facilities, is difficult. Management is often difficult to continue, and communication breakdown with loss of continuity of care is typical. For seamless management to become a reality, the trauma systems need to mature, and new designs for acute trauma clinical reception areas must be developed. Appropriate regionalization of trauma care is needed to concentrate large numbers of severely injured patients in specialized centers with enough volume to allow for specific building designs and provision of appropriate resources, both personnel and equipment, for trauma management.

After initial field assessment, rapid transport will then involve active online communication to the center with coordinated monitoring of vital signs in preparation for patient reception. To streamline patient stabilization, the primary reception area will be staffed by surgical and anesthetic staff who can take the patient through to early definitive care.

As the initial assessment continues, essential radiology and Focused Abdominal Sonogram in Trauma (FAST) will add specificity to the early clinical diagnosis. The integrated clinical reception area will provide the ability to directly roll the patient into the incorporated multi-slice X-ray computed tomography scanner (CAT) or the integrated operating room to allow further essential diagnostic studies or immediate control of failing vital signs.

In patients for whom injury patterns or specific investigations indicate that direct surgical control of hemorrhage is impractical, incorporation of angiographic facilities into the reception/operating suite will allow immediate access to intravascular facilities to restore circulation where practical or stop hemorrhage as needed.

The surgical team can then provide the appropriate initial level of reconstruction before establishing critical care support through to definitive reconstruction and early recovery.

The ability to cool patients to limit tissue damage from underperfusion and secondary injury will be incorporated throughout the entire process. This process is sometimes practiced now and may have a specific role in preserving neurological function and preventing the secondary effects of shock while essential hemostasis and reconstruction occur. In the future, this procedure may well develop into the ability to place the severely injured into a state of organ-preserving life suspension while essential repairs are affected and damaged tissues stimulated to re-grow.

The concept of such streamlined care is not unattainable and is developing in many centers today. Our currently applied standards already require access to coronary angiography within a short period of time to allow early cardiac revascularization for patients presenting with chest pain and ECG ST segment changes. The new operating suite at Massachusetts General Hospital includes rooms with an incorporated CAT scanner, interventional angiography equipment, and an Magnetic resonance imaging scanner (MRI).

The more basic rooms include integrated imaging, allowing multi-screen display of extensive clinical data including vital signs and radiographic data from all sources. While it is interesting to speculate on the coordination of these technical resources, we still believe that the availability of experienced surgical decision-making and interventions requiring high-level surgical skills will remain the most critically important element in patient care.

Despite the development of advanced tools for technology assessment and patient management, the ability to make sound surgical decisions based on basic principles and the historical lessons of trauma surgery will remain the most reliable resource for our patients. Unfortunately, experience has taught us that these lessons have to be relearned with each generation. For example, recent major disasters, such as post-earthquake relief in Haiti and Japan, found surgeons from advanced centers relearning basic skills. Such skills included basic assessment of major injury without the use of advanced technological resources, the value of delayed closure of traumatic amputations, and non-operative management of many simpler fractures.

In the future, complete diagnostics of trauma patients should be achieved within the initial assessment. Currently, many level I trauma centers use initial computer tomography (CT) scanning to assess the main causes of hemorrhage. While this method appears to be accurate for lesions of the trunk, it does not provide information about extremity-related injuries. The patient of the future would certainly benefit from a complete diagnostic assessment, including evaluations of trunk, head, neck, and extremity-related injuries. Ideally, this complete assessment would utilize a technique that requires limited or no radiation and generates data in a rapid fashion.

Moreover, there should be an option to generate 3-D images of specific areas, such as injured articular surfaces and/or soft tissue lesions. This technique would be able to combine the options generated by roentgen techniques and MRI.

In terms of treatment, it will remain key to stop major bleeding immediately. Given the advantages of a damage control concept for life-threatening injuries, alternative techniques may be developed, including catheterization and coiling through minimally invasive techniques. While the minimally invasive techniques are currently time consuming, new options my help to overcome this obstacle. Whether these new options could be used as part of a single approach remains to be seen. For the definitive treatment of major blood loss, there currently appears to be no solution other than the general techniques of surgical management.

For the repair of extremity fractures, the current minimally invasive techniques may still be relevant. It is currently difficult to foresee whether alternative options will be available. For intraoperative assessment during the care of multiply injured patients, new diagnostic options could provide external techniques that do not require blood sampling and yet demonstrate the level of blood loss, the acidity of arterial blood and the level of inflammation. Any new method should be able to be repeated and provide a reliable set of data. Based upon such diagnostic procedures, surgical management can be tapered to minimize the time in intensive care and the hospital stay.

### Total joint registry

The goal of registry studies is to assess population-based outcomes with hospitals and implants of variable standards. According to Benson et al. and Concato et al., well-designed observational studies offer accurate information on treatment effects, and thus, there will be no need to overemphasize the role of randomized controlled studies in clinical decision making [8,9].

Projections predict that the future musculoskeletal patient population will include healthier elderly individuals, individuals with a higher average body weight, and older individuals with better functionality. The early diagnosis of and wider range of treatment concepts for osteoporosis will also result in changes in the future musculoskeletal patient population [10,11].

Trends such as increased life expectancy, the increasing number of elderly patients, and the epidemic of obesity will place an economic burden on total joint arthroplasty. The eighty-five years of age and older age group will be the most rapidly growing age group in the population. Because the prevalence of osteoarthritis of the hip and knee joints is associated with aging, life expectancy and the quality of life will depend on joint replacement surgery [12-14].

Studies addressing registry data have found that, compared to an individual with a normal body mass index, an obese individual is thirty-three times more likely to need a total knee replacement (TKR) [15].

Projections based on data from the Nationwide Inpatient Sample and other sources indicate that the number of total hip arthroplasties will continue to increase gradually, reaching around 500,000 per year in the United States by 2030 [16].

The need for total knee arthroplasty will be much greater [17]. Because patients will outlast their prosthesis, the number of revision arthroplasties will rise accordingly.

As Kurtz et al. indicated, the number of revision arthroplasties has already increased by 79% over the last decade alone. Presently, the failure rate of total hip arthroplasty is 16.9% per year in patients older than sixty-five years [18]. However, according to the Swedish National Hip Arthroplasty Register, the cumulative revision rates for hip and knee arthroplasties increase with younger age [19].

It is difficult to predict the demand for orthopaedic services because of the potential for alternative technologies, the development of new surgical techniques, the use of adjusted algorithms aimed at preventing disease or injury and changes in population demographics, which are dependent on immigration influx.

However, the most obvious fact is that there is a clear reduction in the overall orthopaedic service capacity and health care resources in North America and Europe and an increase in demand for orthopaedic services.

As predicted by Kurtz et al., an increase in the need for primary hip and knee replacement in 2030 of 174% (from 208,600 in 2005 to 572,000 by 2030) and 673% (from 450,000 in 2005 to 3.48 million procedures by 2030), respectively, will occur [20].

A national total joint registry will provide a mechanism to track the longitudinal performance of specific implants of all age groups and may lead to a decrease in the revision burden, as has been the case in Sweden [21].

The revision burden is a percentage calculated by dividing the number of revisions done over a certain period by the total number of primary and revision hip replacements performed during the same time period [21]. For example, in Sweden's registry, the revision burden for cemented prostheses from 1979 to 2000 was 7.4%, while that for uncemented implants was 17.9%. This result supports the hesitation of Swedish surgeons to use uncemented implants. Since 1992, parameters such as patient's age, sex, operated side, diagnosis, implant (manufacturer's catalog number), surgical approach, and cement brand have been registered for primary implants in the Swedish registry [21]. The addition of parameters such as functional demand, body mass index, and physical activity may improve data interpretation [22].

Registries are considered to be a good instrument to improve quality in health care. However, every region has its own population-specific outcome that cannot be compared to other regions' patient outcomes. The addition of information on patient outcomes, satisfaction and radiographic information will improve the impact of the registries worldwide.

The distinct advantage of each country having its own total joint replacement registry is that these registries have the potential to identify complications long before they would be reported in conventional research-based clinical publications. The benefits of having a national guided registry are based on refining indications, surgical techniques, and implant choices [21].

An accurate projection of the number of revision arthroplasties required is needed, as these surgeries involve greater economic resources than primary procedures.

Julin et al. reported the results of a seven-year follow-up study of 32,019 TKRs for primary or secondary osteoarthritis to the Finnish Arthroplasty Register. During the follow-up, 909 TKRs were revised, 205 (23%) due to infection and 704 for other reasons. These data indicate that causes for revision other than infection were more frequent in the 2 younger age groups (81% and 85%) than in the older age group with patients > 65 years of age (74%, p = 0.002), [22]. Because of their higher functional demands, young TKR patients seem to be more likely to require revision [23]. Another group that often undergoes revision is obese TKR patients, and obesity is more common in younger patients compared to older patients who undergo TKR [24,25].

Prosthesis loosening and revision are influenced by the amount of physical activity, that is, the number of steps taken by the patient per year "cyclic loading", and the participation in diverse physical activities. As indicated by Julin et al., "it is not the time in service but the loading of the prosthesis that leads to loosening." They concluded that in the short-term follow-up the relatively young age of ≤ 55 years was accompanied with a higher risk of revision, especially for aseptic failure. With increasing age at the time of surgery, the prosthesis survival rates improved. In the category of 56- to 65-year-old patients, the outcomes were better than in the youngest age group, and outcomes were improved further in patients over 65 years of age. These findings need further clinical assessment [22]. Indeed, in patients who are younger than 55 years old, TKR should only be used in selected cases when there are no other options available, such as Biologic Knee Replacement, i.e., meniscus replacement and articular cartilage paste grafting, to minimize pain and dysfunction.

The National Trauma Data Bank registry that has been available through the American College of Surgeons for a number of years has not been successful because data entry has been voluntary. As one can imagine, neither surgeons nor hospitals are very likely to comply unless absolutely required. Hence, we recommend that Joint Registries should not rely on voluntary based data entry (like the planned American Joint Registry) but rather that it should be managed through the Department of Health, the payors or a similar venue as is the case in Scandinavia.

Some countries like Australia, Canada, and the Scandinavian countries have had great success with registries so far. These registries have served the function of an early warning system for prostheses that fail and have functioned as a good tool for improving the quality of health care. Hence, we advocate for a multinational, government-based organized registry.

### Peri-prosthetic fractures Registry

Peri-prosthetic fractures are considered as a "pre-terminal event" in the majority of patients [26]. The incidence of periprosthetic fractures has more than doubled over a decade period of time [27]. As indicated by Berry et al. [28,29] the worldwide incidence of late periprosthetic femoral fractures is also increasing. Given the increase in total joint arthroplasty, we will encounter an epidemic of periprosthetic fractures that presents a global issue. Thus, optimizing specific algorithms and developing a detailed preoperative management concept that includes specific patient's related parameters, risk factors are needed.

However, distinct algorithms have been developed, there is still a tendency based on surgeon's personal experience whether to perform an osteosynthesis or a revision arthroplasty. Until now there is no consensus on defining the stability of an implant. While, some colleagues focus on x-rays, others prefer to open the proximal femur to assess the hold, and some others converge on the patient's pain history.

Hence, there is a clear need of performing more clinical studies through developing a registry of periprosthetic fractures that may contain detailed information on identifying patients at risk.

In 2006, the Trauma Surgery in Geriatrics group of the German Society for Trauma Surgery established a periprosthetic fracture register within the clinical priority program (CPP) of the AO Foundation (Arbeitsgemeinschaft der Osteosynthese Fragen) to describe the actual standard of treatment and to provide a basis for further improvement. Based on these data, a new classification system needed to be developed that addresses the different aspects of periprosthetic fractures. This classification contains information related to the geriatric population, the status of the prosthesis (e.g., primary or revision arthroplasty), loosening of the prosthesis, cemented or non-cemented fracture location and bone stock (Table 2). The Periprosthetic Fracture Register of the AO Foundation contains following parameters: patient's age, gender, comorbidities, functional demand, body mass index, operated side, diagnosis, implants (manufacturer's catalog number), surgical approach, primary/revision prosthesis, loosening of the prosthesis, cemented versus not cemented, cement brand, location, type of fracture, bone stock, patient's outcome and radiographic information.

**Table 2:** Preliminary Standard treatments based on the CPP of the AO Foundation

Fracture location			
	Around the Stem	Distal the Stem	At the tip of the Stem
**A **Primary			
	**Prosthesis**	**Osteosynthesis**	**Osteosynthesis**

**B **Revision			
	**Prosthesis**	**Osteosynthesis**	**Osteosynthesis**

**C **Loosened Prosthesis			
	**Prosthesis**	**Prosthesis**	**Prosthesis**

**1 **Cemented -**2 **non-cemented			

**1 **Bone quality good - **2 **bad			

Preliminary parameters and standard of treatment that implicates the method based on the CPP of the AO Foundation that is considered as an alpha-numeric code consisting of the letters A-C and numbers 1-3

Several centers retrospectively contributed their cases to a national Periprosthetic Fracture Registry to characterize the current standard of care in these complex clinical cases. In a phase 2 (ongoing process) study, prospective data acquisition will help to develop recommendations to the surgeon and to investigate the outcome in terms of morbidity and mortality, including revision rates and life quality.

Due to the complexity and the heterogeneity of the investigated patient population, a multinational registry seems necessary to prepare for the upcoming demographic changes.

### Quality assurance in orthopaedics

Despite the nationwide implementation of standardized patient safety protocols in the past decade, surgical patients continue being susceptible to preventable complications, including sentinel events and "never events", such as wrong-patient and wrong-site surgery [30,31]. A recent American Academy of Orthopaedic Surgeons (AAOS) survey among 5,540 academy members revealed that 53% of all responders had observed a medical error in the preceding 6 months, including 27 cases of wrong-site surgery [32]. These data emphasize that our current patient safety protocols are indeed not safe in protecting our patients from suffering unintended and preventable harm [33,34]. New strategies to improve patient safety in surgery include the implementation of defined surgical safety checklists, "readbacks" to improve communication in perioperative services, and medical team training programs [35,36].

At the foundation of any program designed to improve patient safety in orthopaedics lies the need for understanding the patterns and root causes of surgical complications. This notion implies the honest and unfiltered reporting, followed by systematic work-up of any reported adverse event in a standardized fashion. However, due to the widespread reluctance to report surgical complications, patient safety in orthopaedics continues to represent an essential "information problem". Disclosing and reporting of medical errors is compelling beyond a doubt from a moral, ethical, and scientific perspective, and therefore represents a basic tenet for improving patient safety.

Underreporting of surgical complications creates a gap of information which may otherwise help prevent the recurrence of a similar adverse event. We have recently described a new Quality Assurance (QA) process in orthopaedics, which was designed to lower the threshold of reporting all perceived complications, "near-misses", and "no-harm events", mandating a standardized peer-review of all reported occurrences in a "real-time" fashion [37]. The new QA process implanted at Denver Health Medical Center relies on the following three cornerstones:

1-Anonymous "real-time" reporting of any suspected adverse occurrence, including "near miss" and "no harm" events, by any member of the surgical team. Occurrences are reported to an independent nurse provider in charge of managing the adverse event database. A "no fault" policy for reporting occurrences is encouraged with strong support from the department leadership.

2-Peer-review of each reported event at a weekly QA conference, using a standardized case review form, in the presence of the responsible attending surgeon and at least two additional faculty staff members who were not involved in the occurrence.

3-Corrective action is defined for each reviewed case, if deemed necessary during the peer-review process. Each closed case is prospectively entered into a departmental QA database. All team members involved in the adverse occurrence are notified about the final assessment of the peer-review process.

**Figure 1:**
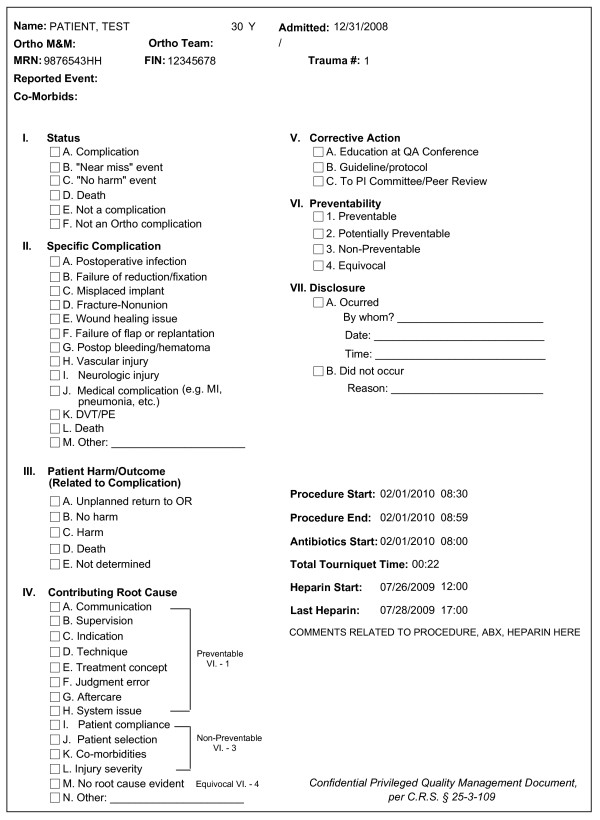
**Case peer-review form for reported adverse occurrences at the Department of Orthopaedics, Denver Health Medical Center (modified version, adapted from Ref #**[37]**)**.

The standardized case review form is shown in Figure 1 impressively, the median rate of reported occurrences increased more than 6-fold from 1.7 to 11.1 per 100 surgical procedures, within 21/2 years of implementation of the new QA process [37]. Similarly, the overall complication rate for the entire Department of Orthopaedics at Denver Health Medical Center increased almost 5-fold, from 1.4% to 6.7%. These data emphasize the "double-edged sword" aspect of reporting adverse events: The reported 5-fold increase in complications within the department likely reflects the improved open and more honest reporting format and critical peer-review of each reported occurrence, rather than a decreased quality of care. And here lies the paradox: If the parameters of "reported adverse events" and "incidence of complications" were used as a measure of institutional quality, the facility would be penalized for its improved surveillance and educational process. The thorough reporting and peer-review of surgical errors creates a new dilemma for the practicing orthopaedic surgeon: an increased quality of reporting leads to an increased complication rate, thus affecting the individual surgeon's professional track record and the respective institution's ranking among peers. Until legislation provides legal protection for medical error disclosure and analysis, we continue to rely on the limited and anecdotal reporting of medical errors and surgical complications in the peer-reviewed biomedical literature [38-40]. Unequivocally, legislative tort reform and avoidance of punitive systems represent the basis required to decrease the surgeons' inherent reluctance to report their own complications.

## Conclusions

This article sheds light on challenges ahead that can only be handled well by logistical preparation that occurs years in advance.

Furthermore, emphasis is placed on developing and improving patient safety protocols in orthopaedics, such as by implementation of standardized quality assurance protocols which are based on open, transparent reporting and peer-review of all adverse events. These challenges require that the international orthopaedic community think more globally and act locally.

## List of abbreviations

IGF2: (insulin-like growth factor 2); TGF-β2: (transforming growth factor beta 2); FGF: (fibroblast growth factor); FAST: (Focused Abdominal Sonogram in Trauma); CAT: (X-ray computed tomography); ECG: (Electrocardiography); MRI: ( Magnetic resonance imaging ); TKR (total knee replacements); AO Foundation: (Arbeitsgemeinschaft der Osteosynthese Fragen); CPP: (clinical priority program); AAOS: (American Academy of Orthopaedic Surgeons); QA: (Quality Assurance); LRP5: (low-density lipoprotein receptor-related protein 5); TNF: (Tumor necrosis factor); IL1:(Interleukin 1); COX2:(Cylooxygenase 2); NF-κB: (nuclear factor 'kappa-light-chain-enhancer of activated B-cells); M&M: (morbidity and mortality); DVT: (deep venous thrombosis); PE: (pulmonary embolism); QA: (quality assurance); PI: (process improvement).

## Competing interests

The authors declare that they have no competing interests.

## Authors' contributions

All authors performed literature review, drafted, and approved the final manuscript.
